# Effectiveness of partial restriction of access to means in jumping suicide: lessons from four bridges in three countries

**DOI:** 10.1017/S2045796024000428

**Published:** 2024-09-18

**Authors:** Sangsoo Shin, Jane Pirkis, Angela Clapperton, Matthew Spittal, Lay San Too

**Affiliations:** Centre for Mental Health and Community Wellbeing, Melbourne School of Population and Global Health, The University of Melbourne, Carlton, VIC, Australia

**Keywords:** bridge, epidemiology, mental health, suicide

## Abstract

**Aims:**

Restricting access to means by installing physical barriers has been shown to be the most effective intervention in preventing jumping suicides on bridges. However, little is known about the effectiveness of partial restriction with interventions that still allow jumping from the bridge.

**Methods:**

This study used a quasi-experimental design. Public sites that met our inclusion criteria were identified using Google search and data on jumping suicides on Bridge A (South Korea), Bridges B and C (the United States) and Bridge D (Canada) were obtained from the relevant datasets. Incidence rate ratios (IRRs) were estimated using Poisson regressions comparing suicide numbers before and after the installation of physical structures at each site.

**Results:**

Fences with sensor wires and spinning handrails installed above existing railings on the Bridge A, and fences at each side of the entrances and the midpoint of main suspension cables on the Bridge D were associated with significant reductions in suicides (IRR 0.37, 95% Confidence Interval (CI) 0.26 *−* 0.54; 0.26, 95% CI 0.09 − 0.76). Installation of bird spike on the parapet on the Bridge B, and fences at the front of seating alcoves on the Bridge C were not associated with changes in suicides (1.21, 95% CI 0.88 − 1.68; 1.49, 95% CI 0.56 − 3.98).

**Conclusions:**

Partial means restriction (such as fences with sensor wires and spinning bars at the top, and partial fencing at selected points) on bridges appears to be helpful in preventing suicide. Although these interventions are unlikely to be as effective as interventions that fully secure the bridge and completely prevent jumping, they might best be thought of as temporary solutions before more complete or permanent structures are implemented.

## Introduction

Suicide is a major public health issue worldwide, accounting for more than 700,000 deaths every year (WHO, 2021). Jumping from height is one means of suicide that warrants particular attention because of the high case fatality associated with attempts, and the impact that these suicides can have on witnesses. Jumping suicide usually occurs at bridges, overpasses, high-rise commercial buildings or natural structures like cliffs which are accessible to the public (Gunnell and Nowers, 1997). It has also been shown to be associated with living in high-rise residences (Lin *et al.*, 2022). These indicate easy access to means may be a contributing factor for jumping suicide. In addition, some jumping sites develop a self-perpetuating reputation as sites where suicides occur, often due to media reporting of deaths at these sites (Ross *et al.*, 2020). The extensive reporting of a suicide with its location information may increase the desirability of the location for suicide (Gross *et al.*, 2007) and create contagion effect (i.e. persons at risk of suicide use the same location for suicide) (Niederkrotenthaler *et al.*, 2012).

To prevent suicides by jumping from bridges, site-based approaches have gradually gained popularity. More specifically, installing barriers to prevent jumps from bridges and similar sites has been shown to be effective in the United States and Canada (e.g., Memorial Bridge in Augusta, Ellington Bridge in Washington D.C., Jacques-Cartier Bridge in Montreal, Bloor Viaduct in Toronto) (Pirkis *et al.*, 2015), leading to an over 80% decrease in jumping suicides at these sites and resulting in minimal displacement to other nearby sites (Dwyer *et al.*, 2023). Current evidence on the effectiveness of physical barriers to prevent jumping suicides at bridges tends to come from studies where the barriers have been of a significant height (ranging from 2 to 5 metres) and have provided full coverage, preventing access to any jumping point (Pirkis *et al.*, 2015).

Less is known about whether barriers are as effective if they are not high or long enough to fully restrict access to the jumping points. The most comprehensive study that has considered this is one conducted in Switzerland (Hemmer *et al.*, 2017). The study looked at suicides before and after the installation of barriers and nets at 15 jumping sites across the country and found that, overall, those with measures that did not secure the whole site were less effective than those that did. Another Norwegian study found, at one bridge, no change of suicide number after partial installation of fence on the part crossing water (Saeheim *et al.*, 2017).

The above-mentioned Switzerland study (Hemmer *et al.*, 2017) used the term *incomplete* for vertical barriers at a bridge, if either of following criteria were met: (1) the head of the bridge was not sealed or (2) the height of the vertical fence was 2 metres or lower. Five of ten bridges with barriers were classified as sites with incomplete measures. However, as concerns about suicides from bridges and the need for barriers has increased, different types of incomplete measures of means restriction on bridges have emerged. These different incomplete measures of access restriction could potentially be applied widely if they reduce suicides. Assessing the effectiveness of relatively lower height barriers and barriers that offer incomplete coverage is important because there are often impediments to the installation of ideal barriers. These impediments include cost and community opposition (Beautrais, 2007). In addition, incomplete measures are sometimes applied as an interim measure.

The current study was designed to provide further insights into the effectiveness of barriers of differing heights and coverage. More specifically, it aimed to (1) evaluate the effectiveness of structures that offer partial restriction of access to means on bridges and (2) inform practical recommendations for those seeking to secure bridges to prevent jumping suicides. We hypothesised that partially restricting access to means on bridges would help in preventing suicide and their impact would vary by the type of partial restriction.

## Methods

### Study design

This study used quasi-experimental design to estimate the impact of partial means restriction at public sites on reducing suicide.

### Definition of partial means restriction

The definition of partial restriction used in a previous study (Hemmer *et al.*, 2017) was modified to capture different forms of restriction. In particular, physical deterrents on the span of the bridge were emphasised. Partial restriction was assumed where the average height of the measure was at eye level of a standing adult, 1.56 m or lower (NASA, 1995), and/or the measure only covered part of the bridge span. As the main interest lay in estimating the effectiveness of newly introduced structures, the sites were excluded if the reason the site was classified as partially restricting access to means was due to pre-existing structural bridge designs (e.g., the presence of points on the bridge that could serve as footholds or handles for climbing).

### Identifying sites and inclusion criteria

A Google search was conducted using the ‘All’, ‘News’ and ‘Images’ sections. The search began with efforts to identify suicides on bridges where barriers had been installed. At this time the search terms were ‘suicide’ AND ‘bridge’. Of the bridges identified by this search, those that satisfied the following criteria were included:

1. The intervention was introduced in response to suicide. This was ascertained from the articles retrieved from the Google search.
2. It was still possible to jump from the main part of the bridge span, even after means restriction. This was ascertained by reviewing each bridge on Google Street View.
3. Pre- and post-intervention data on suicides were available (e.g., data on suicides from government sources like police/sheriff offices or statistics offices). Evidence of this was initially sought from the articles published on Google about the bridge, and then subsequently information was requested from the relevant source or was identified on an official website.

### Site and intervention descriptions

Four bridges with barriers that offered partial restriction of access to means were identified: one in South Korea (Bridge A), two in the United States (Bridge B and C) and one in Canada (Bridge D). Information on the bridges and the measures taken at them is summarized in Table 1 and described in more detail below.

**Table 1.** Profiles of the bridges

Bridge	Location	Applied intervention	Partial restriction
A	South Korea	1.0-metre-high upper fences installed above existing 1.5-metre-high railings on 31 December 2016; with five tension wire sensors and spinning handrail on the top of the upper fence	Jumping possible through space between wires installed at the fence throughout the middle of span
B	The United States	0.1-metre-high bird spike installed on the existing 0.9-metre-high parapet on 1 March 2019	Jumping available over 1.0-metre-high deterrence throughout the span
C	The United States	3-metre-high chain link fences at front of 20 alcoves installed on 31 July 2017, erected on the entire deck on 30 September 2018	Jumping available from none fencing areas neighbouring to the fences around 20 alcoves
D	Canada	Mesh fences with barbed wire on the top at each side of both entrances of the bridge, and at midpoint of main suspender cables installed on 9 November 2011; Upgraded to metal bar fences from old ones at entrances	Jumping available throughout the span except for entrance

Bridge A straddles a river in South Korea and has ten vehicle lanes and footpaths on both sides. Historically, it has been a well-known site for suicide. Following several interventions (e.g., installation of fixed phone boxes providing direct access to a crisis line, CCTV and signage with supportive messages for people in a suicidal crisis), the metropolitan government erected a 1-metre fence over existing 1.5-metre railing, creating a 2.5-metre-hign barrier in late December 2016. The 1-metre upper fence has five tension wire sensors that alert a rescue team if a wire is cut or pulled by more than 10 centimetres. On the top of the upper fence, there are abacus-bead-shaped spinning rails that prevent people gripping the top of the fence to climb over it. There is sufficient space between wire sensors for a person to get their body through, which is the design feature that was considered as offering partial restriction of access to means. In addition, at each entrance, barriers were not installed on the part of the railing that faces the land.

Bridge B is in the United States. It has five vehicle lanes, no footpaths and no shoulder lane. It was built to cross over a bay. The 61-metre-high bridge has long been known as a site for jumping suicides. The regional government body installed 0.1-metre bird spikes on top of an existing 0.9-metre parapet along the bridge on 28 February 2019. The bird spikes are a temporary measure to deter suicidal behaviours until the department installs a more permanent barrier. The spikes are below eye level, and it is still possible to jump from beyond this point, which is why this measure was categorized as only partially restricting access to means of suicide.

Bridge C is also in the United States. It is a historic 45-metre high bridge with two lanes and footpaths on each side. It was built over the land in an urban area. In response to suicides from the bridge, 3-metre chain link fences were installed in front of the 20 seating alcoves on the footpaths at the end of July 2017. During September 2018, these fences were extended along the length of the railings. The period in which the fences were only installed around the seating alcoves was the focus because at this time it was still possible to access jumping points on the bridge.

Bridge D is in Canada. It is a 49-metre high freeway suspension bridge with six lanes and no footpaths, and is located over a river dividing a city into two urban areas. Mesh fences with barbed wire on the top were installed on both sides at each entrance of the bridge on 9 November 2011, at which time barriers were also installed at the midpoints of the main suspension cables. These cables had previously provided an access point for people to climb over to the highest part of the bridge. The barbed wire fences at the entrances were replaced with metal-bar type fences in 2013. No further suicide prevention barriers have been installed on the middle of the span, so this was considered to be partial restriction of access.

### Suicide data sources and period classifications

Table 2 includes the period classifications of each bridge included in this study. For Bridge A, yearly counts of suicides between 1 January 2013 and 31 December 2020 were used, based on figures from an official book published by the Korean Foundation for Suicide Prevention. The figures in the official book were derived from the database of Korean National Investigations of Suicide Victims, which in turn sourced information by reviewing police investigation reports on suicide (see (Na *et al.*, 2019) for more information). For the analysis, 1 January 2013–31 December 2016 was treated as the pre-intervention period and 1 January 2017–31 December 2020 as the post-intervention period.

**Table 2.** Descriptive figures and rate ratio estimates by bridge

	Pre-intervention period		Post-intervention period		
Bridge	Days [Period]	Suicides (per year)	Days [Period]	Suicides (per year)	RR [95% CI]
A	1,461 [1 January 2013–31 December 2016]	102 (25.5)	1,461 [1 January 2017–31 December 2020]	38 (9.5)	0.37 [0.26–0.54]
B	8,094 [1 January 1997–28 February 2019]	209 (9.9)	863 [1 March 2019–30 June 2021]	29 (12.5)	1.30 [0.88–1.92]
C	2,404 [1 January 2011–31 July 2017]	19 (3.4)	426 [1 August 2017–30 September 2018]	5 (4.6)	1.49 [0.56–3.98]
D	4,330 [1 January 2000–8 November 2011]	20 (1.7)	3,341 [9 November 2011–31 December 2020]	<5 (Suppressed)	0.26 [0.09–0.76]

*Note*: 95% CI = 95% confidence interval.

The suicide rate was suppressed as the number of suicides was under 5.

For Bridge B, the dates of each suicide were extracted from a database provided by the medical examiners’ office of a county. The database contained incidents occurring between 1 January 1997 and 31 December 2022. The period 1 January 1997–28 February 2019 was treated as the pre-intervention period and 1 March 2019–31 December 2022 as the post-intervention period.

For Bridge C, a table on suicides was provided by a Police Department, which is responsible for the jurisdiction that includes the bridge. The table included suicides occurring between 1 January 2011 and 22 June 2022. Using an Internet source of information on the installation date (KCAL News, 2018) three time periods were set based on the presence of and extent of covering railing provided by the fencing. Period 1: 1 January 2011–31 July 2017; Period 2: 1 August 2017–30 September 2018 and Period 3: 1 October 2018–22 June 2022. In the main analysis, Period 1 and Period 2 were treated as the pre- and post-intervention periods, respectively. In a supplementary analysis, Period 3 was used to compare the effectiveness of partial- and full-fencing.

For Bridge D, the dates of each suicide between 1 January 2000 and 31 December 2020 were provided by the bureau of the coroner of a state in Canada. The period 1 January 2000–8 November 2011 was treated as the pre-intervention period, and 9 November 2011–31 December 2020 was categorized as the post-intervention period.

### Statistical analysis

Poisson regression analyses were employed to compare suicide rates in the pre- and post-intervention periods at each site. Incidence rate ratios (IRRs) represent the yearly change in rates, with IRRs > 1 indicating an increase in the post-intervention period, and IRRs < 1 suggesting a reduction in the post-intervention period. Analyses were performed using R and package fmsb. The main analyses evaluated the effectiveness of the intervention of interest in preventing suicides. For Bridge B, where the observed time spans between two periods were excessively unbalanced, sensitivity analyses were conducted in which the days of the pre-intervention period were changed. For Bridge C, where fencing was introduced twice with different degrees of coverage, sensitivity analyses were performed by changing the pre- and post-intervention time periods upon each installation. The results of these sensitivity analyses can be found in the supplement file.

### Role of the funding source

There is no specific funding for this study.

## Results

### Suicide number during pre- and post-intervention periods

The number of suicides during the whole study period by bridge and the annual average number of suicides by period were as follows: 140 suicides at Bridge A (on average, 17.5 per year in the whole study period; 25.5 per year in the pre-intervention period and 9.5 per year in the post-intervention period), 238 at Bridge B (10.1 per year in the whole study period; 9.9 per year in the pre-intervention period and 12.5 per year in the post-intervention period), 24 at Bridge C (3.6 per year in the whole study period; 3.4 per year in the pre-intervention period and 4.6 per year in the post-intervention period) and 24 at Bridge D (1.2 per year in the whole study period; 1.7 per year in the pre-intervention period).

### Results from the main analyses

IRRs of suicides for pre- and post- installation periods are presented in Table 2. The rates for Bridge A and Bridge D in the post-intervention period were significantly lower than the rates in the pre-intervention period [IRR for Bridge A = 0.37, 95% Confidence Interval (CI) = 0.26 – 0.54; IRR for Bridge D = 0.26, 95% CI = 0.09 – 0.76]. Rates for Bridge B and C were not significantly different in the post-intervention period and the pre-intervention period (IRR for Bridge B = 1.30, 95% CI = 0.88 – 1.92; IRR for Bridge C = 1.49, 95% CI = 0.56 – 3.98).

### Results from the sensitivity analyses

The models for Bridge B and C were run with different time periods. For Bridge B, when the starting date of the pre-intervention period was changed from 1 January 1997 to 1 January 2000 and 1 January 2010, respectively, the IRRs were 1.22 (95% CI = 0.88 − 1.68) and 0.79 (95% CI = 0.56 − 1.11) (see Supplement 1).

The periods were reclassified in different ways for Bridge C. First, the IRR was 0.83 (95% CI = 0.36 − 1.92) when the time span before the installation of the barrier with full coverage was considered the pre-intervention period and the remainder of the time span was considered the post-intervention period. Second, the IRR was 0.89 (95% CI = 0.37 − 2.12) when the time spans without any barrier and with a fully covered fence were set as pre- and post-intervention periods, respectively (Supplement 2).

## Discussion

This study evaluated the effectiveness of different types of partial means restrictions on bridges in reducing suicide. Partly in line with our expectations, some partial restrictions were effective in preventing suicide. Suicides significantly decreased after installing a fence equipped with sensor wires and spinning rails on top of Bridge A, as well as partial fencing on Bridge D. However, there was no reduction of suicide following partial restriction at other two sites: Bridge B with bird spikes and Bridge C with partial fencing.

The findings from the individual bridges are worth considering in more detail. In the case of Bridge A, the effectiveness is comparable with that of a similar form of intervention on the Clifton Suspension Bridge in the UK, which involved a 2-meter-high wire barrier (Bennewith *et al.*, 2007). However, it is weaker when compared to interventions on bridges with complete restrictions of access to means (e.g., Gateway Bridge in Australia, Grafton Bridge in New Zealand or Ellington Bridge in the US [Pirkis *et al.*, 2015]). So, although the intervention at Bridge A was effective, presumably because the wires and spinning rails made access difficult, it could have been more effective still if the space between the wires had not allowed a person to get through and reach the point of jumping.

On Bridge D in Canada, the partial fencing prevented access to the rails and the cables of the bridge, starting at the entrance. Although the middle part of the span was still accessible, this intervention was effective in preventing suicides. One possible explanation for the positive effect is that it may have increased the likelihood of passers-by being able to intervene with someone at risk. It may have been more difficult to retrieve someone from the cables than from other parts of the bridge, so preventing access to these may have been particularly critical. In addition, the only way to access the middle span of the bridge is via road (because it does not have a pedestrian walkway), and there is no shoulder lane so someone parking on the middle part of the bridge with the intention of jumping would attract the attention of other drivers. There are some indications that those who do jump from bridges are more likely to jump over water than land (Coman *et al.*, 2000), and many suicidal individuals do not show visible signs of distress (Owens *et al.*, 2019), which may make recognition difficult (Owens *et al.*, 2019) without these other signs of unusual behaviour. By-standers are more likely to assist in situations where the risk of danger is evident (Fischer *et al.*, 2011; Ngo *et al.*, 2022), so if the suicidal individual’s behaviour was more noticeable this may contribute to timely intervention. On the other hand, the highest part of the bridge remained accessible at 49 metres, so it is likely that a more complete intervention that involved full coverage of the bridge would have been more effective still.

The bird spikes on Bridge B had the advantage of being light weight and not obstructing the view but were not effective in reducing suicides, irrespective of how the pre-intervention period was defined. Similarly, the partial fencing on Bridge C was not effective in preventing suicides, but once fences with fuller coverage were erected the result was much more positive [Supplement 2]. This could be attributed to the similarity in design between the railing areas and the alcove areas, which may potentially enable individuals to readily access these areas and use them as footholds for jumping.

The current study provides important information about the effectiveness of partial restriction of access to jumping points on bridges and considers a broader set of interventions that has not been considered in the past. It is also the first study to consider partial means restriction on bridges in more than one country. However, the study has several limitations. Official data on suicides at the bridges in question may be imprecise (Beautrais, 2001) and the fact that suicide is a rare event limits the power of the statistical analyses (Reisch and Michel, 2005; Sinyor and Levitt, 2010). This latter limitation is important because some of the interventions that did not show statistical evidence of effectiveness may in fact have been promising. Another limitation is that the bridges in the current study might not represent many other sites where similar interventions are put in place. Finally, unmeasured confounders may also have been a limitation; it was not possible to ascertain, for example, what other factors may have been influencing suicide rates in the local area, or whether other interventions were being delivered.

## Conclusions

Partial restriction of access to means on bridges appears to be helpful in preventing suicide, in certain circumstances. Fences with sensor wires and spinning bars at the top, and partial fencing at selected points on bridges show promise, although further work is required to verify these findings. Although these interventions are unlikely to be as effective as interventions that fully secure the bridge and completely prevent jumping, they might best be thought of as temporary solutions before more complete or permanent structures are implemented.

## Supporting information

Shin et al. supplementary material Shin et al. supplementary material

## Data Availability

We concealed the names of included sites to mitigate the imitation of suicidal behaviour (use the sites for suicide) from readers who are at risk of suicide. However, we will provide the names of sites and data providers, as well as information on data access availability, upon reasonable request.

## Supplementary material

The supplementary material for this article can be found at https://doi.org/10.1017/S2045796024000428.

## References

[ref1] Beautrais A (2001) Effectiveness of barriers at suicide jumping sites: A case study. *Australian & New Zealand Journal of Psychiatry* 35(5), 557–562. 11551268 10.1080/0004867010060501

[ref2] Beautrais A (2007) Suicide by jumping - A review of research and prevention strategies. *Crisis-the Journal of Crisis Intervention and Suicide Prevention* 28, 58–63. 10.1027/0227-5910.28.S1.58 26212196

[ref3] Bennewith O, Nowers M and Gunnell D (2007) Effect of barriers on the Clifton suspension bridge, England, on local patterns of suicide: Implications for prevention. *British Journal of Psychiatry* 190, 266–267. 10.1192/bjp.bp.106.027136 17329749

[ref4] Coman M, Meyer ADM and Cameron PA (2000) Jumping from the Westgate Bridge, Melbourne. *Medical Journal of Australia* 172(2), 67–69. 10738475 10.5694/j.1326-5377.2000.tb139202.x

[ref5] Dwyer J, Spittal MJ, Scurrah K, Pirkis J, Bugeja L and Clapperton A (2023) Structural intervention at one bridge decreases the overall jumping suicide rate in Victoria, Australia. *Epidemiology and Psychiatric Sciences* 32, e58. 10.1017/S2045796023000720 PMC10539743 37721170

[ref6] Fischer P, Krueger JI, Greitemeyer T, Vogrincic C, Kastenmuller A, Frey D, Heene M, Wicher M and Kainbacher M (2011) The bystander-effect: A meta-analytic review on bystander intervention in dangerous and non-dangerous emergencies. *Psychological Bulletin* 137(4), 517–537. 21534650 10.1037/a0023304

[ref7] Gross C, Piper TM, Bucciarelli A, Tardiff K, Vlahov D and Galea S (2007) Suicide tourism in Manhattan, New York City, 1990-2004. *Journal of Urban Health-Bulletin of the New York Academy of Medicine* 84(6), 755–765. 17885807 10.1007/s11524-007-9224-0 PMC2232032

[ref8] Gunnell D and Nowers M (1997) Suicide by jumping. *Acta Psychiatrica Scandinavica* 96(1), 1–6. 9259217 10.1111/j.1600-0447.1997.tb09897.x

[ref9] Hemmer A, Meier P and Reisch T (2017) Comparing different suicide prevention measures at bridges and buildings: Lessons we have learned from a national survey in Switzerland. *PLoS One* 12(1), e0169625. 10.1371/journal.pone.0169625 PMC5218568 28060950

[ref10] KCAL News (2018) So-Called ‘Suicide Bridge’ In Pasadena to get permanent, taller barriers. https://youtu.be/zQy1Ez3E9oc?si=w6a9Uc7ePXIrbQVW (accessed July 20 2023).

[ref11] Lin CY, Hsu CY, Chen YY, Chang SS and Gunnell D (2022) Method-specific suicide rates and accessibility of means a small-area analysis in Taipei City, Taiwan. *Crisis-the Journal of Crisis Intervention and Suicide Prevention* 43(5), 375–384. 10.1027/0227-5910/a000793 PMC9578364 34003021

[ref12] Na EJ, Choi J, Kim D, Kwon H, Lee Y, Lee G, Fava M, Mischoulon D, Jang J and Jeon HJ (2019) Design and methods of the Korean national investigations of 70,000 suicide victims through police records (the KNIGHTS study). *Psychiatry Investigation* 16(10), 777. 10.30773/pi.2019.07.14 PMC6801314 31455061

[ref13] NASA (1995) 3 Anthropometry and biomechanics. Available at https://msis.jsc.nasa.gov/sections/section03.htm (accessed May 10 2024).

[ref14] Ngo NV, Gregor SD, Beavan G and Riley B (2022) The role of bystanders in the prevention of railway suicides in New South Wales, Australia. *Crisis-the Journal of Crisis Intervention and Suicide Prevention* 43(5), 412–418. 10.1027/0227-5910/a000804 PMC9578362 34405696

[ref15] Niederkrotenthaler T, Sonneck G, Dervic K, Nader IW, Voracek M, Kapusta ND, Etzersdorfer E, Mittendorfer-Rutz E and Dorner T (2012) Predictors of suicide and suicide attempt in subway stations: A population-based ecological study. *Journal of Urban Health-Bulletin of the New York Academy of Medicine* 89(2), 339–353. 22318375 10.1007/s11524-011-9656-4 PMC3324611

[ref16] Owens C, Derges J and Abraham C (2019) Intervening to prevent a suicide in a public place: A qualitative study of effective interventions by lay people. *BMJ Open* 9(11), e032319. 10.1136/bmjopen-2019-032319 PMC6887022 31740473

[ref17] Pirkis J, Too LS, Spittal MJ, Krysinska K, Robinson J and Cheung YTD (2015) Interventions to reduce suicides at suicide hotspots: A systematic review and meta-analysis. *The Lancet Psychiatry* 2(11), 994–1001. 26409438 10.1016/S2215-0366(15)00266-7

[ref18] Reisch T and Michel K (2005) Securing a suicide hot spot: Effects of a safety net at the Bern Muenster Terrace. *Suicide and Life-Threatening Behavior* 35(4), 460–467. 16178698 10.1521/suli.2005.35.4.460

[ref19] Ross V, Koo YW and Kolves K (2020) A suicide prevention initiative at a jumping site: A mixed-methods evaluation. *EClinicalMedicine* 19, 100265. 10.1016/j.eclinm.2020.100265 PMC7046509 32140675

[ref20] Saeheim A, Hestetun I, Mork E, Nrugham L and Mehlum L (2017) A 12-year national study of suicide by jumping from bridges in Norway. *Archives of Suicide Research* 21(4), 568–576. 27309998 10.1080/13811118.2016.1199988

[ref21] Sinyor M and Levitt AJ (2010) Effect of a barrier at Bloor Street Viaduct on suicide rates in Toronto: Natural experiment. *British Medical Journal*, 341. 10.1136/bmj.c2884 PMC2897976 20605890

[ref22] WHO (2021) Suicide. https://www.who.int/news-room/fact-sheets/detail/suicide (accessed February 21 2024).

