# Supplementary material for: Effectiveness of partial restriction of access to means in jumping suicide: lessons from four bridges in three countries
Source: Epidemiol Psychiatr Sci. 2024 Sep 18;33:e38. doi: 10.1017/S2045796024000428 (PMC11450421; doi:10.1017/S2045796024000428)
Supplement: Shin et al. supplementary material [file S2045796024000428sup001.docx]

Supplement 1: Descriptive statistics and incidence rate ratio estimates by different cut-off of pre-intervention period, Bridge B, United States

| Pre-intervention Period | | Post-intervention Period | | RR  [95% CI] |
| --- | --- | --- | --- | --- |
| Days  [Period] | Suicides | Days  [Period] | Suicides |  |
| 8,094  [Jan 1 1997 – Feb 28 2019] | 209 | 1,402  [Mar 1 2019 – Jun 30 2021] | 44 | 1.22  [0.88 - 1.68] |
| 6,999  [Jan 1 2000 – Feb 28 2019] | 193 | 1,402  [Mar 1 2019 – Jun 30 2021] | 44 | 1.14  [0.82 - 1.58] |
| 3,346  [Jan 1 2010 – Feb 28 2019] | 133 | 1,402  [Mar 1 2019 – Dec 31 2022] | 44 | 0.79  [0.56 – 1.11] |

Note) RR = Rate Ratio, 95% CI = 95% Confidence Interval.

Supplement 2: Descriptive statistics and incidence rate ratio estimates by different cut-off of pre-intervention period, Bridge C, United States

| Pre-intervention Period | | Post-intervention Period | | RR  [95% CI] |
| --- | --- | --- | --- | --- |
| Days  [Period] | Suicides | Days  [Period] | Suicides |  |
| 2,404 (Period 1)  [Jan 1 2011 – July 31 2017] | 19 | 426 (Period 2)  [Mar 1 2019 – Sep 30 2019] | 5 | 1·49  [0.55 - 3.98] |
| 2,830 (Period 1 + 2)  [Jan 1 2011 – Sep 30 2019] | 24 | 996 (Period 3)  [Oct 1 2019 – Jun 22 2022] | 7 | 0·83  [0.36 – 1.92] |
| 2,404 (Period 1)  [Jan 1 2011 – July 31 2017] | 19 | 996 (Period 3)  [Oct 1 2019 – Jun 22 2022] | 7 | 0.89  [0.37 - 2.12] |

Note 1) RR = Rate Ratio, 95% CI = 95% Confidence Interval.

Note 2) Period 1 refers to time span without fence, period 2 refers to time span with partial fences at alcoves and period 3 refers to time span with fully covering fence on the bridge.
